# Anxiety–metabolism networks in human anorexia nervosa: Responses to caloric and non-caloric meals in the Networks Integrating Anxiety and Metabolism in Anorexia Nervosa (NAMA) randomized trial

**DOI:** 10.1017/S0007114526106448

**Published:** 2026-06-28

**Authors:** Erik Ekbäck, Lal Yalçin, Özgün Özalay, Gabriel Granåsen, Burcu Özbaran, Ali Saffet Gönül, Olof Lagerlöf

**Affiliations:** 1 Department of Clinical Sciences, Umeå University, Umeå, Sweden; 2 Department of Public Health and Clinical Medicine, Umeå University, Umeå, Sweden; 3 Wallenberg Centre for Molecular Medicine, Umeå University, Umeå, Sweden; 4 Department of Psychiatry, Ege University, Izmir, Turkey; 5 Munroe-Meyer Institute, University of Nebraska Medical Center, Omaha, USA; 6 Department of Child and Adolescent Psychiatry, Ege University, Izmir, Turkey; 7 Department of Medical Translational Biology, Umeå University, Umeå, Sweden

**Keywords:** Anorexia nervosa, Randomised controlled trial, MRI, Study protocol, Food intake

## Abstract

Anorexia nervosa (AN) is an eating disorder that is mediated by psychological and metabolic factors, yet it is unclear how these factors interact. The NAMA trial objective is to clarify the metabo–psychiatric interaction and identify how it affects AN patients’ behaviour. This randomised trial will recruit thirty-six treatment-naïve female AN patients, 13–18 years of age, and thirty-six matched healthy controls. Participants will undergo psychiatric assessments followed by 12-h overnight fasting. The next morning, baseline assessments of outcomes will be performed. Patients will be randomly allocated 1:1 to receive a mixture with calories or receive a mixture without calories. Healthy controls will also be allocated to receive mixtures with/without calories. Mixtures will be standardised for taste and appearance, and allocation will be masked. The primary outcome measure is resting-state functional MRI 60 min post-consumption of the mixture. Secondary outcomes include (1) blood samples to study markers reflecting metabolic states, hunger/satiety and stress responses, (2) psychometric evaluations of subjective experiences and (3) assessment, in a second meal 3 h later, of the effects of previous calorie intake on subsequent food consumption. This article describes the study protocol, including the analysis plan, for a randomised controlled trial to comprehensively evaluate the effects of calorie intake in AN. The trial will distinguish psychological and metabolic neuronal networks associated with food intake and uncover how their integration affects food intake and other hallmark symptoms in AN. The aim is to accelerate treatment development by identifying brain mechanisms that drive AN.

Patients with anorexia nervosa (AN) fear gaining weight and have a distorted body image and low body weight resulting from insufficient food intake^(1)^. AN has a devastating effect on the lives of affected individuals and a markedly high mortality^(2,3)^. Onset peaks in adolescence^(4–6)^. Adolescents, at least in Western countries, often internalise a thin-body ideal^(7–9)^. Large case–control studies have associated AN with environments emphasising dieting as well as other social and personal contexts suggested to make young individuals psychologically vulnerable to AN^(9–14)^. However, many of these contextual factors are non-specific and common to other psychiatric disorders^(13)^. Additionally, most young individuals exposed to thin-body ideals do not develop AN^(15)^. Several prospective studies have failed to identify thin-body ideal internalisation and other psychological factors related to pursuing thinness as risk factors for AN^(15–18)^. Rather, it has been shown that negative affect or neuroticism and perfectionism predict AN^(12,17,19,20)^. Psychotherapy together with nutritional rehabilitation can treat AN, yet patients frequently relapse, and remission typically takes years to achieve if achieved at all^(3,21,22)^. Hence, it is unclear how psychological factors specifically affect the development and maintenance of AN^(15,23,24)^.

Emerging evidence suggests that psychological factors may interact with metabolism to precipitate AN^(25–27)^. Well-powered genetic studies have recently shown that a large part of AN heritability can be explained by a genetic architecture mediating psychiatric and metabolic traits^(28–30)^. While few AN genes have been identified so far, several of those identified are associated with metabolic signalling^(28,31–33)^. This is in line with psychometric data showing that AN patients typically feel more satiated than healthy individuals when eating food^(34–36)^. Additionally, decreasing nutrient availability by inhibiting glycolysis pharmacologically made patients with AN feel satiated, not hungry, as it did for healthy controls^(37)^. To distinguish unspecific psychological reactions from the metabolic signalling effects of nutrients, a gastric load experiment blinded AN patients and healthy controls to receive either glucose or water through a nasogastric tube. The more glucose AN patients received, the more they experienced a dose-dependent exaggerated satiety, higher fear of being fat and marked activation of the corticotropic stress pathway^(38)^. Although in AN most peripheral hormonal pathways respond as expected to a low nutritional state and reverse upon weight gain, these and other observations indicate that AN is associated with a perturbed response to metabolic signals of food intake^(25,39)^.

The perturbed response to metabolic signals in AN is associated with altered brain activity^(40–42)^. Genes linked to AN are functionally enriched for expression predominantly in brain neurones^(28,43)^. Neurones in the hypothalamus constitute a major metabolic control hub for caloric intake^(44)^. The hypothalamus has been shown to react differently to both blinded gastric loading with glucose and sucrose tasting in AN^(42,45)^. Feeding reduces the hypothalamic levels of the intracellular metabolites glutamate and glutamine in AN patients. This is paradoxical as feeding instead increases them in normal weight and constitutionally lean individuals^(46)^. Chemogenetic activation of the metabolically regulated agouti-related peptide-expressing neurones in the hypothalamus can ameliorate the development of activity-based anorexia, a mouse model of voluntary self-starvation and other hallmark AN characteristics^(47)^. AN also affects the processing of metabolic stimuli in brain areas critical for reward and emotional regulation, such as the amygdala and ventral striatum, as well as parts of the cortex mediating cognitive aspects of food intake^(41,42,45,48–50)^. Functional connectivity analyses indicate that AN perturbs how different brain areas communicate in response to metabolic fluctuations^(27,42,45,46)^. This effect on how the brain processes metabolic fluctuations by integrating information from metabolic, emotional-motivational and cognitive brain areas is supported by neurocircuit mapping in rodents showing that the decision to eat is not a function of discrete areas but rather their intercommunication^(51)^. Metabolic state affects brain processing of various stimuli in patients with acute AN as well as weight-recovered individuals, indicating that how AN affects the brain response to metabolic fluctuations is part of the core AN pathology and not just a result of weight loss^(40,41,49,50)^.

While these observations indicate that neuronal networks in the brain integrate metabolic and psychological aspects of eating differently in AN, it is unclear how this perturbed metabo–psychological integration contributes to AN development and maintenance^(52)^. No previous report has, to our knowledge, revealed in what way the connection between psychology and metabolism mediates core symptoms of AN; that is, no one has distinguished either what neuronal networks respond to the psychological *contra* metabolic aspects of eating a meal or how those networks integrate their information to affect patients’ food intake, distorted body image and fear of weight gain. The identification of the mechanisms that drive AN is also hampered by the current tendency of published studies to focus on adults rather than adolescents although adolescence is the time when most patients develop AN. The ISRCTN and Clinicaltrials.gov registers (as of October 2025) record neither any recent nor ongoing trials anywhere in the world aimed to answer these questions. No current clinical trials of AN comprehensively speak to metabo–psychological mechanisms of change.

The primary objective of the Networks Integrating Anxiety and Metabolism in Anorexia Nervosa (NAMA) trial is to distinguish between the neural response to eating caloric and non-caloric meals and how this differs between newly diagnosed adolescents with AN and matched healthy controls. Secondary objectives examine the effects of caloric and non-caloric food intake on endocrine, circulatory, psychological and behavioural responses in patients with AN and healthy controls. By integrating behavioural, psychological and physiological aspects of food intake with brain network analyses, the NAMA trial will investigate what drives AN. We hypothesise that calorie-dependent changes in network activity will differ between AN patients and healthy controls and that calorie intake will predict aggravated AN-related symptoms in patients: lower food intake, worse fear of gaining weight and increased feeling of being overweight.

The NAMA trial is designed to distinguish psychological and metabolic neural networks in the brain, uncover how these are associated with food intake and explain how their integration affects food intake and other hallmark symptoms of AN. This is, to our knowledge, the first masked clinical trial studying how the metabo–psychological interaction drives the disease in first-onset, treatment-naïve patients. The NAMA trial’s ultimate aim is to accelerate treatment development for AN based on clinically integrated pathophysiological research.

## Experimental methods

### Design

The study is designed as a single-centre, parallel-group randomised controlled trial with two study arms: (1) calorie intake in meal-1 and (2) no calorie intake in meal-1. Participants and healthy controls are individually randomised to either one of the two arms, both delivered at the eating disorder clinic of the Ege University Hospital, Faculty of Medicine.

The primary outcome measure is brain patterns observed with functional MRI, and secondary outcomes include endocrine markers in blood, cardiovascular responses, self-reported symptom severity and subsequent food consumption.

The study is planned following the Standard Protocol Items: Recommendations for Interventional Trials^(53,54)^ and will be analysed and reported following the recommendations in Consolidated Standards of Reporting Trials^(55)^, with special care taken to meet extended recommendations for both trials with patient-reported outcomes^(56)^ and non-pharmacological trials^(57)^.

### Protocol design and conduct

This is the study protocol version 1.0, 2025-05-10. For details on the trial registration, see Table 1. To ensure transparency and integrity throughout the study, all changes or deviations from the protocol will be thoroughly documented and reported. Any modifications to the protocol, including changes to study objectives, design, participant population, sample size, procedures or significant administrative aspects, will necessitate a formal protocol amendment. Similarly, any alterations that could potentially impact participant safety, benefits or risks will be carefully evaluated and documented. Proposed amendments will be reviewed and approved by the study team and submitted for approval to the national ethical review board before implementation. Additionally, all relevant study personnel will be promptly informed of these changes to ensure compliance and consistency across the study.

**Table 1.** Trial registration data Table 1 long description.

Data category	Information
Primary registry and trial identifying number	ClinicalTrials.gov NCT06814002
Date of registration in the primary registry	2025-02-06
Primary sponsor	Umeå University, Ege University
Contact for public and scientific queries	Olof Lagerlöf
Public title	NAMA trial
Scientific title	Networks integrating anxiety and metabolism in anorexia nervosa trial
Countries of recruitment	Turkey
Health condition studied	Anorexia nervosa
Interventions	Meal with 400 kcal and meal without calories
Key inclusion and exclusion criteria	Inclusion criteria: female sex, 13–18 years of age, patient at the eating disorder clinic, AN DSM-5 diagnosis, treatment naïve. Healthy controls are accepted (eating disorder in the present or past is an exclusion criterion for them).
	Exclusion criteria: chronic medical conditions, medications that affect hormone levels or eating behaviours, severe psychiatric disorders other than AN, drug abuse, pregnancy, high risk of refeeding syndrome, allergies to provided meal content, cognitive impairment, contraindications for MRI, fasting blood glucose above 6·0 mmol/l on the morning of the study
Study type	Interventional
	Allocation: randomised
	Primary purpose: mechanistic research
Date of first enrolment	2025-02-15
Target sample size	72
Recruitment status	Recruiting
Primary outcome	MRI
Key secondary outcomes	Food consumption, psychometric scales, blood samples, blood pressure, pulse

AN, anorexia nervosa, DSM-5, Diagnostic and Statistical Manual of Mental Disorders, 5th edition, kcal, kilocalorie.

### Recruitment

The study started to recruit participants in February 2025, and the last measurements for the primary manuscript are expected to be collected by the end of 2026.

Recruitment, interventions and data collection will be performed in the specialised eating disorder clinic of the Faculty of Medicine at Ege University Hospital in Izmir (population approximately 4·5 million), Turkey. Before participants are recruited, we will ensure the implementation of Good Clinical Practice guidelines, the establishment of a functioning clinical research infrastructure and the training of study staff.

Participants will be recruited through four possible pathways: (1) AN patients will be recruited from Ege University Faculty of Medicine Hospital by physicians meeting these patients. Healthy controls will be recruited through (2) announcements on Ege University campuses, (3) local hospitals and (4) online platforms such as Instagram.

Individuals who agree to participate will be contacted by study personnel for further information about the study over the telephone. At that time, an initial assessment for eligibility will be performed. For further eligibility assessments, please see the next heading. For adolescents who cannot be reached over the telephone, parents or legal guardians will be contacted. Potentially eligible participants will be scheduled to a study physician for further eligibility assessment and written informed consent.

### Eligibility

Female individuals of 13–18 years of age who are currently patients at the eating disorder clinic and newly diagnosed with AN according to the Diagnostic and Statistical Manual of Mental Disorders, 5th edition^(58)^, and treatment naïve will be eligible. Healthy control participants will be matched on age, sex and education level with the AN participants. The clinical diagnosis/diagnoses for participants will be validated/ruled out by the Schedule for Affective Disorders and Schizophrenia for School-Age Children – Present and Lifetime version^(59)^. The presence of other exclusion criteria will also be further explored at the clinical visit.

1. The exclusion criteria are as follows:
2. Having one or several chronic medical conditions that may interfere with or hinder participation or affect study outcomes.
3. Use of medications that affect hormone levels or eating behaviours.
4. Having one or several severe psychiatric disorders other than AN (e.g. schizophrenia, major depressive disorder, bipolar disorder).
5. Current drug abuse, pregnancy or potential pregnancy (for AN patients, these conditions are excluded as a part of routine care. For healthy controls, no drug testing will be performed.).
6. High risk of refeeding syndrome or severe complications related to eating disorders.
7. Inability to comply with the nutritional programme of the study.
8. Allergies to foods included in the provided meal content.
9. Cognitive impairments preventing comprehension of the study or the informed consent process.
10. Contraindications for MRI (e.g. pacemaker, prosthesis, claustrophobia)
11. Fasting blood sugar above 6·0 mmol/l on the morning of the study, as this indicates either non-compliance with overnight fasting or pre-diabetes/diabetes.

#### Additional exclusion criterion for healthy controls

(1) Having one or several eating disorders, current or past.

Unexpected findings of biochemical anomalies at baseline (please see ‘Assessments’) will not be considered exclusion criteria, and medical conditions will be referred for appropriate treatment.

Participant inclusion in the study will be determined by a study clinician based on the outlined eligibility criteria. In cases of uncertainty, the principal investigator will make the final decision. Importantly, to maintain impartiality and avoid selective recruitment bias, enrolling clinicians will be masked to future treatment allocations, as randomisation will occur at a later point in time. Participants will receive both oral and written information about the study and will provide written informed consent before inclusion. For participants below 18 years of age, written informed consent will also be obtained from parents or legal guardians. Participation in the study is voluntary, and participants may withdraw at any time without any negative consequences or affected subsequent care. Participants may also choose to withdraw any personal data already collected.

### Randomisation

When an eligible participant has been recruited and consented, they will be scheduled for randomisation at a later timepoint. AN participants will be individually randomised to one of the two study arms with a 1:1 allocation ratio by a computer-generated allocation sequence using permuted blocks. The block sizes will vary randomly between two and four.

Randomisation lists will have been pre-prepared by a separate researcher who is not involved in participant recruitment. Sealed envelopes will contain the study ID for each participant and the meal assigned to that individual (caloric or non-caloric). When a participant is ready to enter the study, a researcher will draw an envelope and prepare the assigned meal for that participant in the evening for consumption the next morning.

Participants with AN will be admitted to the clinic’s open inpatient unit on the evening prior to study participation to ensure a 12-h overnight fast. On the following day, they will receive the specified interventions and undergo the outlined assessments. Once an AN participant has been randomised and completed the study, a matched healthy control participant will be recruited. Recruitment of healthy control participants will be conducted by study personnel who are masked to the randomisation outcomes of the corresponding AN participant. This approach ensures that the allocation of both AN and healthy control participants remains concealed from recruiting researchers and those conducting assessments. Healthy control participants will undergo the same eligibility assessments as AN participants, be assigned their own study ID and share the same allocation as their corresponding AN participant. However, unlike AN participants, healthy control participants will not be admitted overnight.

Codes will be used to increase information confidentiality and participant anonymity, and participant allocation will be performed based on these codes. Randomisation will continue until *n* 36 AN patients and *n* 36 healthy controls have completed the study as per-protocol participants.

The randomisation results will not be revealed to anyone other than the individual preparing the assigned meal. The randomisation step will thus be separated from the patient enrolment process and all the other processes of the study. Through that, participants, study personnel and clinicians delivering any co-interventions will be masked to participants’ allocation and so will outcome-assessors and statistical analysts. Participants will be asked to guess their personal allocation after meal-1 to enable a validation of the blinding integrity.

### Intervention protocol

Depending on their randomisation result, participants will be asked to consume one of two jellies, both composed by a dietitian and prepared by study personnel: one containing 400 kcal and the other calorie-free. Both participants and the person handing out the jelly will be unaware of its caloric content. Participants will be given 15 min to consume the jelly, without forcing them to do so, and the amount consumed will be recorded.

The caloric food used in the study will be prepared using gelatine powder, water, granulated sugar, cinnamon, ginger, turmeric and grated lemon zest. To prepare the mixture, plain gelatine powder and 80 g of granulated sugar are mixed with 100 ml of hot water until a homogeneous consistency is achieved. For flavouring, one teaspoon each of cinnamon, ginger and turmeric, along with the zest of half a lemon, is added. The mixture is then brought to a boil, and after 5 min of boiling, the heat is turned off, and the mixture is left to cool for 15 min. It is then poured into a single-serving container, allowed to cool at room temperature and placed in the refrigerator for 2–3 h to solidify. The final product weighs 250 g.

The ingredients of the calorie-free food are water, agar-agar, two teaspoons of the natural sweetener Stevia, cinnamon, ginger, turmeric, grated lemon zest and lemon juice. As with the preparation of the caloric food, all ingredients are mixed and brought to a boil. After it starts boiling, it is stirred for five more minutes, and once it starts to thicken, the heat is turned off, and the mixture is left to cool for 15 min. The prepared meal is then poured into a single-serving container and refrigerated for 2–3 h until it is ready to be consumed. The final product weighs 250 g.

The ingredients and preparation methods were carefully chosen to ensure that both mixtures have a similar appearance and taste.

Three hours after consuming the first mixture (meal-1), participants will be offered a second meal (meal-2). This meal will always contain calories and consist of a nutritionally balanced, standard oatmeal dish with a distinct taste from the first meal. Participants will be allowed to eat freely, up to a maximum of 500 kcal, to minimise the risk of medical complications such as refeeding syndrome in participants with AN. Participants will have 45 min to complete the meal, and the total amount consumed will be recorded.

Apart from meal-1 and meal-2, any oral intake apart from water will be prohibited during the time of the study.

### Adherence to study protocol

Protocol non-adherence is defined as follows:

1. A participant withdraws their consent, choosing to discontinue the study from the time of randomisation to the end of T_2_ data collection.
2. A participant is discovered to have received the wrong type of meal in meal-1.
3. A participant consumes ≤ 30 % of the food provided in meal-1.
4. A participant eats or drinks anything other than the provided meals and water between T_0_ and T_2_ data collection.
5. A participant refuses or is unable to undergo MRI scanning at T_2_.
6. A participant is unable to adhere to the study schedule.
7. An adverse event occurs that renders continued participation inappropriate. Please see ‘Adverse events and security plan’ for details.

All outcome measures detailed under ‘Assessments’ will be collected from all participants. For the primary analysis, all data from participants adhering to the protocol will be included. For details, see the ‘Statistical Methods’ section.

### Adverse events and security plan

Adverse events (AE) are defined as any undesirable experiences requiring healthcare that occur to a participant during the study, regardless of whether they are related to the study or not. All AE reported spontaneously by participants or their relatives or observed by investigators, study personnel or clinicians will be documented in the electronic case report form. Both AE and serious AE – including those resulting in death, being life-threatening, requiring hospitalisation or involving other significant medical concerns – will be promptly reported to the principal investigator. Serious AE that are assessed as potentially related to the study will be reported to the accredited ethics committee that approved the protocol within 7 d of the responsible researcher becoming aware of the event.

Based on previous studies and unpublished preliminary data, significant deterioration of symptoms during the study is not anticipated. However, for safety purposes, self-report questionnaires collected throughout the study will be reviewed by study personnel who will also monitor participant distress. Patients are expected to become anxious when asked to eat a meal, and regular meal-related symptom fluctuations will not be considered as AE. Any other observed worsening of symptoms or adverse reactions will be addressed in discussions with the participant and, when appropriate, their parents or legal guardians.

As the interventions and assessments are provided as part of the regular healthcare system, all participants are covered under standard patient injury insurance. External independent study monitoring will be performed to ensure that all data storage, handling and analysis procedures comply with ethical guidelines, regulatory requirements, quality standards and protocol.

### Assessments

Pre- and post-meal self-report scales will be administered via a secure online platform accessible only to the research team. Pseudonymised data from these scales, as well as all other assessments, will be stored on a secure server with regular security backups. Biological specimens collected during the study will be coded and stored in coolers in a certified biobank for batchwise analysis after the dataset is complete. Physical records will be stored in locked cabinets with restricted access. The pseudonymisation key will be securely stored in a location that is separate from the data.

Participants’ travel expenses and food and beverage costs will be covered, and this amounts to approximately 20 US dollars. Those who fail to attend scheduled appointments as a part of the study will be reminded via telephone or email. Parents and/or legal guardians may also be contacted. A detailed schedule of enrolment, interventions and assessments is presented in Table 2.

**Table 2.** Schedule of enrolment, interventions and assessments Table 2 long description.

	Study period					
	Enrolment			Post-allocation		
Timepoint	T_-1_	T_0_	Allocation	T_1_ (post-meal, 15 min)	T_2_ (1 h)	T_3_ (3 h)
Enrolment						
Eligibility screening	X					
Informed consent	X					
BMI calculation	X					
Allocation			X			
Interventions						
Calorie intake in meal-1			I 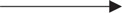	X		
No calorie intake in meal-1			I 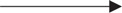	X		
Assessments						
Meal-1 food consumption				X		
Self-report	X	X		X	X	X
Blood sampling		X			X	
Blood pressure and pulse		X			X	
MRI		X			X	
Meal-2 food consumption						X

### Eligibility measures

The medical records of all participants will be analysed to identify comorbidities and other exclusion criteria. The biochemical results that will be available for AN patients include lipid profile (total cholesterol, HDL-cholesterol, LDL-cholesterol and TAG levels), blood glucose level, insulin level, thyroid-stimulating hormone, T4, follicle-stimulating hormone, luteinising hormone, oestradiol, prolactin and C-reactive protein. These parameters are routinely checked on all AN patients. No additional blood tests will be performed to identify exclusion criteria, and for healthy controls, these blood tests will not be performed.

Clinician interviews will be performed to verify the clinical AN diagnosis in patients and to rule out any comorbidity in AN and healthy control participants. The Schedule for Affective Disorders and Schizophrenia for School-Age Children – Present and Lifetime version will be used to complement the clinical interview, and it will be performed by a well-trained psychiatrist used to applying the instrument in practice. The Schedule for Affective Disorders and Schizophrenia for School-Age Children – Present and Lifetime version is a reliable and valid tool for diagnosing psychiatric disorders^(59)^.

### Assessments for description of sample

Body weight and height will be measured at T_-1_ to calculate BMI. At the same time, the participants’ sociodemographic background will be assessed using a brief self-made questionnaire. Additionally, at T_-1_, the following self-rating scales will be collected for screening purposes and description of sample: (1) the Autism Spectrum Screening Questionnaire^(60,61)^, (2) the Childhood Trauma Questionnaire^(62)^, (3) Children’s Depression Inventory^(63)^, (4) the Screen for Child Anxiety Related Disorders^(64)^, (5) the Eating Attitudes Test^(65)^ and (6) the Global Assessment of Functioning scale^(66)^.

### Outcome measures

Outcome measures will be assessed at baseline before randomisation (T_0_), 15 min after meal-1 (T_1_), 1 h after meal-1 (T_2_) and 3 h after meal-1 (T_3_), with some measures collected only at specific timepoints. Details for each outcome measure are provided under their respective subheadings and summarised in Table 2. If a participant misses a scheduled data collection timepoint, the following steps will be taken in sequence: (1) if feasible and meaningful, the data will be collected as soon as the issue is identified; (2) if not, the data point may be recorded as missing, and the participant will continue in the study; or (3) if the participant is deemed no longer relevant to the study, they will be excluded from further follow-up and per-protocol analysis.

#### Primary outcome measure

##### MRI

Resting-state MRI scans will be performed at T_0_ and T_2_. MRI examinations will be conducted using a 3.0 Tesla MRI scanner (Siemens Magnetom Verio, Numaris/4, Syngo MR B17) equipped with a 12-channel head coil. First, structural images will be obtained with axial TSE T2-weighted, coronal 3D-SPACE FLAIR and 3D-MPRAGE sequences. Structural images will be reviewed by an on-site radiologist to identify any brain anomalies or pathologies that would render a potential participant ineligible (e.g. structural abnormalities, lesions). If no pathological findings are detected, imaging will continue with a functional MRI sequence, including 9 min of resting-state functional MRI, where participants will keep their eyes open and look at a fixation cross on screen.

#### Secondary outcome measures

##### Self-report

At T_0_, T_1_, T_2_ and T_3_, psychometric evaluations will be performed on Visual Analogue Scales. The following questions will be used at the appropriate timepoints:

1. How anxious are you about consuming the meal? Response options: I feel no anxiety – I feel very anxious.
2. How often do you feel anxious about eating unfamiliar foods? Response options: I do not feel anxious – I feel very anxious.
3. How hungry do you feel? Response options: I feel full – I feel very hungry.
4. How worried are you about the possibility of gaining weight from consuming the meal? Response options: I feel no anxiety – I feel very anxious.
5. How much do you fear becoming obese? Response options: I do not fear – I fear a lot.
6. How much does the thought of becoming obese affect your eating habits? Response options: It does not affect at all – It affects a lot.
7. How overweight do you feel? Response options: I feel very thin – I feel very overweight.

##### Blood sampling

At T_0_ and T_2_, blood samples will be collected from all participants. At T_0_, participants’ fasting glucose will be measured. Neuroendocrine measurements on blood samples taken at T_0_ will include ghrelin, obestatin, peptide YY, leptin and cortisol hormones. These analyses will be conducted using available kits. At T_2_, the same neuroendocrine parameters will be analysed in blood.

##### Blood pressure and pulse

To evaluate cardiovascular responses, blood pressure and heart rate measurements will be performed using an automatic blood pressure monitor and a pulse oximeter (both Sun Tech Medical) at T_0_ and T_2_.

##### Meal-1 and meal-2 food consumption

Although not strictly considered an outcome, the amount of mixture (in grams) consumed in the meal-1 will be recorded and included as a control variable in the analysis. The amount of food consumed in meal-2 will also be recorded by weighing the plate before and after the meal.

### Statistical methods

#### Analysis plan

Analysis will be conducted once data collection is complete. No interim analyses will be performed. Initially, the quality and completeness of the data will be assessed. If available, missing values will be entered using information from the individual’s original data file. The dataset will be examined for outliers and inconsistencies. For categorical variables, any responses outside the defined categories will be coded as missing. For continuous variables, values outside the acceptable ranges will also be coded as missing. Missing data will not be imputed.

The analyses will be carried out by the investigators and a biostatistician using the latest version of SPSS Statistics (IBM Corp.), R (R-core team) or equivalent statistical software. For the analysis of functional MRI data, additional software tools described below will be used. Data analysts will be blinded to the participants’ allocation. Significance testing will be two-tailed, and statistical uncertainties will be reported as 95 % confidence intervals. All tests will be conducted with a significance level of 0·05.

#### Descriptive statistics

Baseline data and descriptive statistics will be reported using standard measures in accordance with Consolidated Standards of Reporting Trials guidelines, and the baseline characteristics of the included participants will be reported per randomisation group and AN status in a baseline table. The following baseline characteristics will be reported: age (years), AN subtype (by categories), co-morbidity (% yes, by diagnostic categories), smoking (% yes, frequency), country of birth (% Turkey, % first-generation immigrant and % second-generation immigrant – with at least one parent born outside of Turkey). Sex will not be reported as all participants will be of female sex.

#### Patient flow diagram

**Figure 1.**
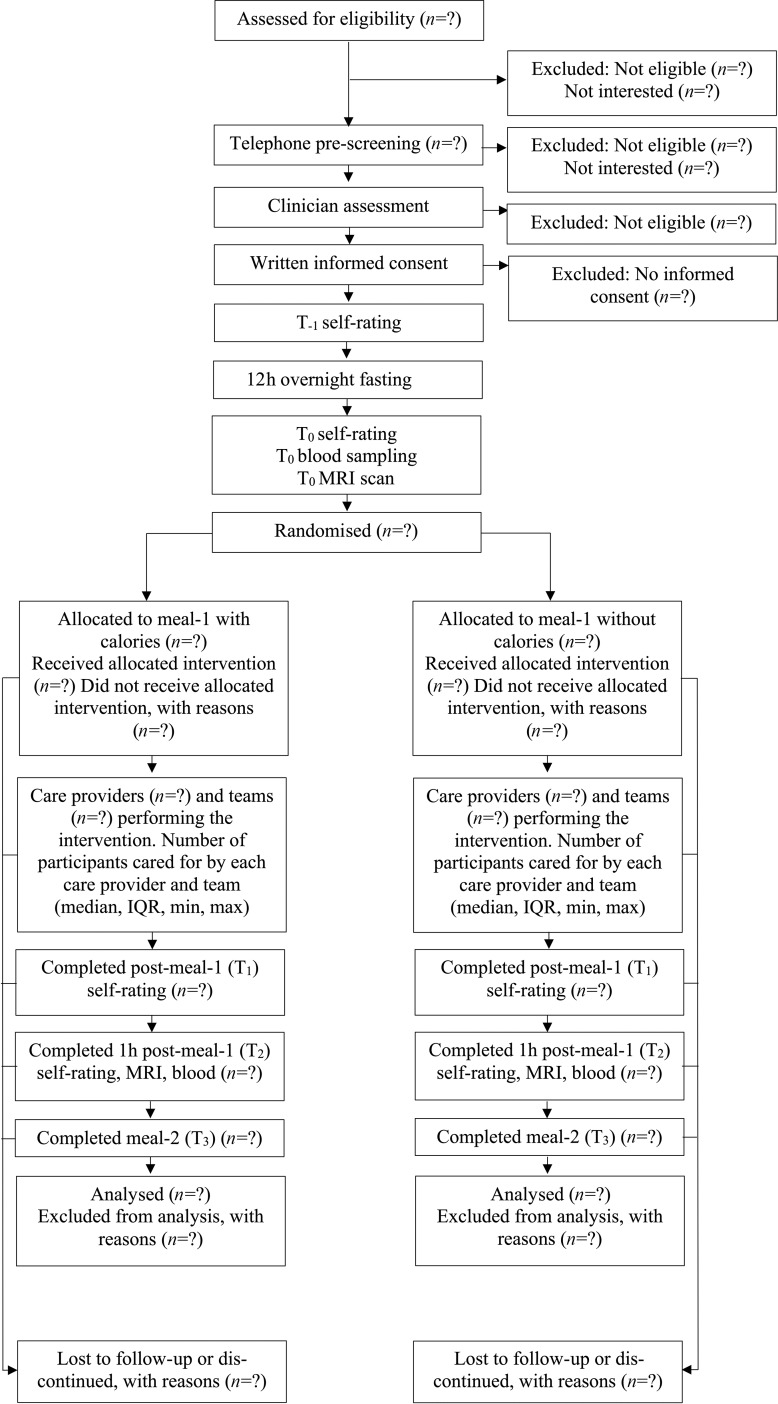
Flow of participants.

The flow of participants will be illustrated in a flow diagram according to Consolidated Standards of Reporting Trials recommendations (Figure 1).

#### Primary analysis

For the statistical analysis of functional neuroimaging data, MATLAB and Statistical Parametric Mapping (SPM12) software will be used to assess brain activity patterns and differences across groups. Connectivity analysis will be performed using the CONN Toolbox to examine the relationships between different brain regions and networks. Neuroimaging data will first undergo pre-processing to ensure the data is suitable for further statistical analysis.

The pre-processing steps that will be performed include the following:

1. Realignment: This is the process of correcting head motion of the participants.
2. Slice-Time Correction: This involves correcting for functional differences between slices acquired every 3 s.
3. Coregistration: Anatomical and functional images are overlaid to determine the anatomical location of activation.
4. Spatial Normalisation: This process accounts for individual brain shapes by normalising anatomical MRI images. The template developed by the Montreal Neurological Institute will be used.
5. Smoothing: This is used to improve image quality and will be performed using a 3D Gaussian kernel (full width at half maximum = 8 mm).

After the pre-processing steps, the data of all participants will be registered to the common Harvard–Oxford Cortical Atlas space. Participant and group-level regression analyses will be performed in this atlas space. Functional connectivity analyses will be performed between regions of the components of the Salience Network, including the anterior insula, dorsal anterior cingulate cortex, temporoparietal junction and hypothalamus. Changes in the topology of networks formed by these four regions, in addition to their functional connectivity with each other, will be analysed using Graph Theory. The changes in the connectivity features of these regions before and after caloric/non-caloric intake will be evaluated using both nodal and global metrics. Significant results obtained will be recalculated using masks transferred back to the participant space. Beta values of the regression analysis will be controlled for type-1 errors with false discovery rate and reported accordingly.

The independent variables of the regression analyses will include (1) type of meal (caloric or non-caloric), (2) AN status (AN or healthy control), (3) AN subtype (restrictive or bulimic type), (4) BMI and (5) duration of AN illness. A random effect for each participant and for each matched set of patient and healthy control will be used to account for within-subject correlations from repeated measurements and dependencies within each matched pair.

#### Secondary analyses

Secondary outcome variables will be modelled using similar regression models as used in the primary analysis. In these models, time (T_0_/T_1_/T_2_) and the allocation by time interaction will be included as fixed effects, and a random intercept for each subject will be included when analysing variables that are measured at more than two timepoints. Additional parametric tests will be performed as long as the assumptions for them are met. Depending on the type of data, the following statistical methods will be used: Independent *t* test, Pearson *r* and ANOVA. Where necessary, corresponding non-parametric alternatives will be used.

#### Dose–response analyses

Additional dose–response analyses will be performed by controlling the previously specified regression models for the amount of mixture consumed in meal-1.

#### Sample size calculation

A two-tailed power calculation for 70 % power and *P* = 0·05 was performed using G * Power software, for a *t* test with linear regression (two independent groups) and an effect size of d = 0·6, based on results from similar neuroimaging studies^(42)^, determining that thirty-four participants per group (AN/healthy) would be sufficient. To account for potential dropouts or unforeseen issues, thirty-six participants will be recruited for each group to ensure robust statistical analysis.

## Discussion

The NAMA trial will be the first randomised controlled trial in AN that distinguishes between psychological and metabolic neural networks in the brain. The trial is designed to uncover how these networks are associated with food intake and explain how their integration affects food intake and other hallmark symptoms of AN. Recruitment of patients and healthy controls, intervention delivery and data collection started at the beginning of 2025, and the last follow-up that will be presented in the primary manuscript is expected to be completed in 2026. The present article presents the overall methods of the experiment and the main analyses that are planned to be performed. It was written to prepare for future analyses and to increase the transparency of scientific conduct, as well as to enable others to comment on our proposed strategy. In this way, the results will be as robust and transparent as possible.

AN is a severe mental disorder for which available treatments are far from satisfactory^(67)^. This is partially due to the scarcity of clinically integrated mechanistic experiments. It is, for example, unclear how psychological factors affect the development of AN^(15,23,24)^, and emerging evidence suggests that psychological factors interact with metabolism to precipitate AN^(25–27)^. AN is associated with a perturbed response to metabolic signals of food intake^(25,39)^, and this is associated with altered brain activity^(40,42)^. No previous report has clarified how this perturbed metabo–psychological integration contributes to AN development and maintenance^(52)^.

The design of the NAMA trial makes it possible for the first time to reveal how the integration of metabolic and psychological factors drives AN pathology by performing a two-meal behavioural test with multiple assessments of brain network activity, physiological parameters and psychometric data. A single study on AN rarely collects neuroimaging data, blood samples, physiological parameters and psychometric data. Masking participants to oral intake of food with and without calories has, to our knowledge, not been performed by anyone in the past. This is expected to activate metabolic responses in a way that is closer to the natural biological reactions to food intake than feeding through a nasogastric tube. The NAMA trial is also original in focusing on treatment-naïve adolescents with AN. Treatment-naïve adolescents with AN are an understudied group that is expected to shed much-needed light on the mechanisms of AN, as adolescence is the period in which disease onset peaks^(4–6)^. In addition, the inclusion of healthy controls will enable researchers to dissect the distinct mechanisms in patients in a way that is disease specific. Unlike most other AN studies aimed to reveal disease mechanisms, using the randomised controlled trial method enables masking, which reduces many common biases^(55)^. The randomised controlled trial method is also considered the most rigorous ‘gold-standard’ way of testing experimental medical interventions^(68)^. With such a broad approach, the NAMA trial will make it possible to uncover the mechanisms that drive AN in ways that were previously impossible.

Through the integration of multifaceted data from patients and healthy controls, the NAMA trial may thus change the way we understand the metabolic effects of calorie intake in health and disease and in particular what distinguishes the physiology of AN patients from that of healthy individuals. This may open the door to new therapeutic alternatives and change how patients with AN are seen and treated. Indeed, the view of AN as resulting from psychological processes that induce dieting does not account for the amount of recent data that suggest that AN develops out of an interaction between psychological and metabolic factors^(28,69)^, and this may have delayed targeted drug development for this disorder.

The present study should be assessed with some limitations in mind. First, the analysis plan has purposefully been formulated somewhat loosely, and in so doing, it is not possible to take full advantage of one of the main reasons for methodological pre-specification: to prevent selective outcome reporting. The field of neuroimaging in general and neuroimaging of AN in particular evolves quickly, and it is expected that in 2 years, there will be both new methodological approaches to analysis, particularly new machine learning algorithms, and new neuroimaging studies of AN that need to be considered will have been published. Additionally, we are performing parallel *in vitro* and rodent experiments that will inform the region-of-interest selection and analysis. To enable such translational synergistic effects, it was considered necessary to formulate the analysis plan in this way. All the other purposes of pre-specifying a research strategy are still fulfilled^(70)^, and results will be assessed based on biological feasibility. Second, it is anticipated that some participants with AN will be unwilling to consume the full amount of mixture prepared in meal-1 or to consume meal-2. This may result in reduced contrast between study arms; however, it is not expected to bias the results with type-1 errors. The mixtures were designed to be small enough for ease of consumption yet large enough to produce a metabolic effect and to recruit newly diagnosed patients with AN, which is expected to facilitate the intake, as these individuals are unlikely to have advanced disorders. Third, it is unclear to what extent the masking of participants will be successful, as the approach is still somewhat novel. Even if some unmasking occurs, the study may be able to detect important differences between participants with AN and healthy controls, although the individual effects of psychological and physiological processes will not be fully disentangled. Apart from the participants, all individuals involved in the study are expected to be successfully masked. Finally, the trial evaluates neuroimaging, physiological, subjective and behavioural outcomes with a short follow-up time. Given the limited intervention involved, there is no intention to treat participants, and therefore, no clinical outcomes or hard endpoints are included.

### Conclusion

The NAMA trial will distinguish psychological and metabolic neural networks in the brain, uncover how these are associated with food intake and explain how their integration affects food intake and other hallmark symptoms of AN. This study will thereby reveal neurobiological mechanisms driving AN.

## Table 1 Long description

A table summarizing trial registration data for a study on anorexia nervosa. The table has 16 rows and 2 columns. The columns are labeled 'Data category' and 'Information'. Row 1: Primary registry and trial identifying number, ClinicalTrials.gov NCT06814002. Row 2: Date of registration in the primary registry, 2025-02-06. Row 3: Primary sponsor, Umeå University, Ege University. Row 4: Contact for public and scientific queries, Olof Lagerlöf. Row 5: Public title, NAMA trial. Row 6: Scientific title, Networks integrating anxiety and metabolism in anorexia nervosa trial. Row 7: Countries of recruitment, Turkey. Row 8: Health condition studied, Anorexia nervosa. Row 9: Interventions, Meal with 400 kcal and meal without calories. Row 10: Key inclusion and exclusion criteria, Inclusion criteria: female sex, 13-18 years of age, patient at the eating disorder clinic, AN DSM-5 diagnosis, treatment naïve. Healthy controls are accepted (eating disorder in the present or past is an exclusion criterion for them). Exclusion criteria: chronic medical conditions, medications that affect hormone levels or eating behaviours, severe psychiatric disorders other than AN, drug abuse, pregnancy, high risk of refeeding syndrome, allergies to provided meal content, cognitive impairment, contraindications for MRI, fasting blood glucose above 6.0 mmol/l on the morning of the study. Row 11: Study type, Interventional. Row 12: Allocation, randomised. Row 13: Primary purpose, mechanistic research. Row 14: Date of first enrolment, 2025-02-15. Row 15: Target sample size, 72. Row 16: Recruitment status, Recruiting. Row 17: Primary outcome, MRI. Row 18: Key secondary outcomes, Food consumption, psychometric scales, blood samples, blood pressure, pulse.

Navigate back to Table 1.

## Table 2 Long description

A table detailing the schedule of enrolment, interventions, and assessments. The table is divided into several columns representing different timepoints: T-1, T0, Allocation, T1 (post-meal, 15 min), T2 (1 h), and T3 (3 h). The rows are categorized into Enrolment, Eligibility screening, Informed consent, BMI calculation, Allocation, Interventions, Assessments, Meal-1 food consumption, Self-report, Blood sampling, Blood pressure and pulse, MRI, and Meal-2 food consumption. Row 1: Enrolment, no values. Row 2: Eligibility screening, X under T-1. Row 3: Informed consent, X under T-1. Row 4: BMI calculation, X under T-1. Row 5: Allocation, X under Allocation. Row 6: Interventions, Calorie intake in meal-1, arrow from T0 to T1. Row 7: Interventions, No calorie intake in meal-1, arrow from T0 to T1. Row 8: Assessments, Meal-1 food consumption, X under T0. Row 9: Assessments, Self-report, X under T-1, T0, T1, T2, T3. Row 10: Assessments, Blood sampling, X under T-1, T1, T2, T3. Row 11: Assessments, Blood pressure and pulse, X under T-1, T1, T2, T3. Row 12: Assessments, MRI, X under T-1, T3. Row 13: Assessments, Meal-2 food consumption, X under T3.

Navigate back to Table 2.
